# Sleeptalking! Sleepwalking! Side Effects of Montelukast

**DOI:** 10.1155/2013/813786

**Published:** 2013-09-05

**Authors:** Samer Alkhuja, Natalya Gazizov, Mary Ellen Alexander

**Affiliations:** The Commonwealth Medical College, Pocono Medical Center, 175 East Brown Street, Suite 203, East Stroudsburg, PA 18301, USA

## Abstract

A 16-year-old Caucasian female presented to the pulmonary clinic for a followup on her asthma. Due to the worsening of allergy-related symptoms, therapy with montelukast 10 mg daily was started and resulted in good relief of the patient's symptoms. In the nights following initiating therapy with montelukast, the patient's mother reported daily parasomnias in the form of sleeptalking and sleepwalking. Montelukast was discontinued, and that resulted in absence of the parasomnias. In a second attempt montelukast was reinstituted to control the patient's symptoms. Parasomnias were immediately reported after resuming therapy. Montelukast was then discontinued indefinitely. Our patient has never had any history of parasomnias, and since the discontinuation of montelukast, parasomnias were never reported again. Parasomnias in the form of sleeptalking or sleepwalking were not previously reported as adverse effects of montelukast. Alternative modalities to treat allergy-related symptoms in patients, who develop parasomnias while receiving montelukast, should be explored.

## 1. Introduction

A 16-year-old Caucasian female presented to the pulmonary clinic for a followup on her asthma which has been treated with fluticasone propionate 250 mcg/salmeterol 50 mcg and albuterol sulfate inhalers. Past medical history included asthma and allergic rhinitis. There were no reported symptoms related to obstructive sleep apnea (OSA), and no history of any psychological disorders. There was no family history of sleepwalking, sleeptalking, or other forms of parasomnias. Due to the worsening of allergy-related symptoms (ARS), including allergic rhinitis, therapy with montelukast 10 mg daily was started and resulted in good relief of the patient's ARS. In the nights following initiating therapy with montelukast, the patient's mother reported daily parasomnias in the form of sleeptalking and sleepwalking. Montelukast was discontinued, and that resulted in absence of the parasomnias. A few days later, montelukast was reinstituted in a second attempt to control the patient's ARS; however, sleeptalking and sleepwalking were reported again immediately after resuming therapy. Montelukast was discontinued indefinitely. Our patient has never had any history of parasomnias, and since the discontinuation of montelukast, parasomnias were never reported again. The application of Naranjo scale (Table 1) revealed a score of eight, indicating a probable adverse drug effect [1]. The use of the World Health Organization-The Uppsala Monitoring Center (WHO-UMC) system for standardized case causality assessment revealed a causality term of “certain” (Table 2) [2].

## 2. Discussion

Parasomnias in the form of sleepwalking and sleeptalking are common. In a review of behavior-related experiences in clinical trials of montelukast by Philip et al., the overall behavior-related adverse experiences were infrequent [3], with only three serious adverse effects reported (anxiety disorder, depression, and schizoaffective disorder), resulting in a frequency of 0.03%, and all were in adults [3]. 

Bygdell et al. analyzed 744 psychiatric adverse reactions among a total of 600 individual case safety reports concerning children in Sweden and found that montelukast was one of the three most frequently suspected drugs which resulted in psychiatric adverse drug reactions [4]. A wide range of reactions were reported including aggressiveness which was reported more frequently for boys than for girls, as were suicidal conditions [4]. 

Furthermore, Wallerstedt et al. reviewed 48 reports of psychiatric disorders in children during treatment with montelukast. Psychiatric disorders reported more than once included nightmares, unspecified anxiety, aggressiveness, sleep disorders, insomnia, irritability, hallucination, hyperactivity, and a personality disorder [5]. Parasomnias in the form of sleeptalking and sleepwalking were not reported as part of the sleep disorders by Wallerstedt et al. [5]. The time from exposure to the development of an adverse drug reaction was less than one week [5].

Children with OSA, if not treated, frequently exhibit neurocognitive and behavioral morbidities and may benefit from treatment with leukotriene (LT) modifier therapy drugs such as montelukast [6]. Montelukast has been used to treat ARS; however, its use was reported by Goldbart et al. to reduce the apnea hypopnea index (AHI) in children with OSA [4]. The reduction in AHI was more than 50% in 65.2% of the treated children [7]. 

Goldbart et al. performed quantitative polymerase chain reaction, immunohistochemistry, and Western blotting for gene and protein expression of LK receptors (LK1-R) and (LK2-R) and for concentrations of LTB4 and LTC4/D4/E4 in adenoid and tonsillar tissues from children with OSA or recurrent throat infections [6]. They found increased LT1-R and LT-2R protein expression and higher levels of LTB4 and LTC4/D4/E4 in tissue taken from children with OSA [6]. The improvement in sleep was attributed to the effect of LTs modifier therapy in exhibiting dose-dependent reduction in adenotonsillar cellular proliferation rates and adenoid size [6, 8]. 

HLA and genetic susceptibilities to sleepwalking were reported by Lecendreux et al. [9]. Twenty-one sleepwalkers were found to be DQB_1_ ∗ 0501 positive verses eight controls [9]. The family data for all HLA subtypes were further assed, and a significant excess transmission was observed for DQB_1_ ∗ 05 and ∗04 alleles in familial cases, strongly suggesting that specific DQB1 genes are implicated in disorders of motor control during sleep [9]. Since the HLA genes help in marking freign cells, which in turn facilitate them being attacked by the immune system, Lecendreux et al. speculated that sleepwalking might be due to the immune system targeting cells important for sleep regulation [9].

The possible mechanism of this side effect of montelukast could be due to its effect on the cysteinyl leukotriene type 1 (CysLT1) receptor in the central nervous system by being a CysLT1 receptors antagonist. This speculation is supported by the review by Wang et al. of the role of CysLT1 receptors in the central nervous system related to behavior and the effects of montelukast as a CysLT1 receptors antagonist [10]. However, Wang et al. also mentioned that potential effects of montelukast might occur as a result of off-target activities of the drug [10].

## 3. Conclusion

Parasomnias in the form of sleeptalking or sleepwalking were not previously reported as adverse effects of montelukast. Sleeptalking is an event of utterances to coherent conversation during sleep, and it is of little medical concern [11]. Sleepwalking events are partial arousals occurring during the first half of sleep [11]. Sleepwalking can be minor or elaborate behaviors including even driving [11]. Although montelukast reduces AHI in patients with OSA by treating upper airway ARS [6], its use may result in the development of parasomnias. In such an event, the discontinuation of montelukast is usually sufficient to relieve this adverse effect. Alternative modalities to treat ARS in patients who develop parasomnias while receiving montelukast should be explored. Those possible alternatives may include a higher dose of inhaled corticosteroids in asthma, topical nasal corticosteroids or nasal antihistaminic drugs for rhinitis, or the use of omalizumab.

## Figures and Tables

**Table 1:** 

Naranjo algorithm				
	Yes	No	Do not know or not done	Our patient
(1) Are there previous conclusive reports on this reaction?	+1	0	0	0
(2) Did the adverse events appear after the suspected drug was given?	+2	−1	0	+2
(3) Did the adverse reaction improve when the drug was discontinued or a specific antagonist was given?	+1	0	0	+1
(4) Did the adverse reaction appear when the drug was readministered?	+2	−1	0	+2
(5) Are there alternative causes that could have caused the reaction?	−1	+2	0	+2
(6) Did the reaction reappear when a placebo was given?	−1	+1	0	0
(7) Was the drug detected in any body fluid in toxic concentrations?	+1	0	0	0
(8) Was the reaction more severe when the dose was increased, or less severe when the dose was increased?	+1	0	0	0
(9) Did the patient have a similar reaction to the same or similar drugs in any previous exposure?	+1	0	0	0
(10) Was the adverse event confirmed by any objective evidence?	+1	0	0	+1

Totals				8

>9: definite adverse drug reaction.

5–8: probable adverse drug reaction.

1–4: possible adverse drug reaction.

0: doubtful adverse drug reaction.

**Table 2:** 

WHO-UMC causality categories		
Causality term	Assessment criteria	Yes/no
Certain	Event or laboratory test abnormality, with plausible time relationship to drug intake.	Yes
	Cannot be explained by disease or other drugs.	Yes
	Response to withdrawal plausible (pharmacologically, pathologically).	Yes
	Event definitive pharmacologically or phenomenologically (i.e., an objective and specific medical disorder or a recognized pharmacological phenomenon).	yes
	Rechallenge satisfactory, if necessary.	Yes

	Final outcome	Certain
